# Laparoscopic complete mesocolic excision with true central vascular ligation for right-sided colon cancer

**DOI:** 10.1007/s00464-020-07867-z

**Published:** 2020-08-19

**Authors:** Masanobu Enomoto, Kenji Katsumata, Kenta Kasahara, Tomoya Tago, Naoto Okazaki, Takahiro Wada, Hiroshi Kuwabara, Junichi Mazaki, Tetsuo Ishizaki, Yuichi Nagakawa, Akihiko Tsuchida

**Affiliations:** Department of Gastrointestinal and Pediatric Surgery, Tokyo Medical University, 6-7-1 Nishi-Shinjuku, Shinjuku-ku, Tokyo, 160-0023 Japan

**Keywords:** Complete mesocolic excision, D3 lymph node dissection, Laparoscopy, Colon cancer, Right hemicolectomy

## Abstract

**Background:**

Complete mesocolic excision (CME) is known to be effective for colon cancer. However, in right-sided colon cancer, central vascular ligation (CVL) is not easy to perform. In particular, in patients in whom the superior mesenteric vein (SMV) runs on the ventral side of the superior mesenteric artery (SMA) (Type V/A), laparoscopic ligation of the artery at its root is extremely difficult compared with this procedure in patients in whom the SMA runs on the ventral side of the SMV (Type A/V).

**Methods:**

We started performing laparoscopic CME with true CVL for right-sided colon cancer using the SMA as a landmark in 2015, and by 2019, we had completed it for 60 patients. To start, the mesocolon is opened well to the caudal side of the ileocolic vessels. The mesentery is then fully detached from the retroperitoneal tissue, after which the ileocolic vessels are ligated at their roots. D3 lymph node dissection of the lymph nodes around the SMA and SMV on the resection side is also performed using the SMA as a landmark, and depending on the location of the tumor, the roots of the right and middle colic vessels are ligated and divided. This study was conducted with the approval of the Tokyo Medical University Ethics Committee. All patients provided informed consent.

**Results:**

The tumor was located in the cecum in 21 cases, the ascending colon in 33, and the transverse colon in 6. The mean operating time was 229 min and the mean volume of hemorrhage was 67 ml. There was one Clavien-Dindo Grade 3 or worse postoperative complication (ileus). There were no surgery-related or in-hospital deaths.

**Conclusion:**

This procedure can be performed comparatively safely. However, since it requires some skill, we consider that it should only be performed in suitable cases by teams with sufficient experience.

**Electronic supplementary material:**

The online version of this article (10.1007/s00464-020-07867-z) contains supplementary material, which is available to authorized users.

The effectiveness of complete mesocolic excision (CME) for colon cancer is commonly known [1, 2]. CME is generally performed with the aim of separating the mesocolon from the parietal plane to allow true central ligation of the supplying arteries and draining veins at their roots [1]. However, in right-sided colon cancer, the courses of the vessels concerned are complex, and central vascular ligation (CVL) is not easy to perform. In particular, in patients in whom the superior mesenteric vein (SMV) runs on the ventral side of the superior mesenteric artery (SMA) (Type V/A), laparoscopic ligation of the artery at its root is extremely difficult compared with this procedure in patients in whom the SMA runs on the ventral side of the SMV (Type A/V). Consequently, in almost all Japanese hospitals, mesocolic excision is performed using the left margin of the SMV as a landmark and without ligating the root of the artery, which contradicts the aim of performing CME. Although a previous report showed laparoscopic lymph node dissection for right-sided colon cancer using the SMA as a landmark without video [3] and another showed the same using the SMV as a landmark with a video [4], no video of this procedure with the SMA as a landmark has been published.

## Materials and methods

We started performing this operation in 2015, and by 2019, we had completed it for 60 patients. We have not performed it for patients with metastases in lymph nodes around the SMA diagnosed on the basis of preoperative investigations, performing open surgery in such cases.

Our dissection of the lymph nodes around the SMA and ligation and division of the vessels at their roots while using the SMA as a landmark during laparoscopic complete mesocolic excision with true central vascular ligation for right-sided colon cancer are presented. To start, the mesocolon is opened well to the caudal side of the ileocolic vessels. The mesentery is then fully detached from the retroperitoneal tissue, after which the ileocolic vessels are ligated at their roots. D3 lymph node dissection of the lymph nodes around the SMA and SMV on the resection side is also performed using the SMA as a landmark, and depending on the location of the tumor, the roots of the right and middle colic vessels are ligated and divided. This study was conducted with the approval of the Tokyo Medical University Ethics Committee. All patients received an explanation of the procedure and provided informed consent.

## Results

The tumor was located in the cecum in 21 cases, the ascending colon in 33, and the transverse colon in 6. The courses of the SMA and SMV were Type A/V in 27 cases and Type V/A in 33. Three patients underwent another surgical procedure during the same operation; the mean operating time for the remaining 57 patients was 229 min (range 118–414 min), and the mean volume of hemorrhage was 67 ml (range 0–770 ml). Intraoperative complications consisted of damage to the middle colic vein in one case and to the accessory right colic vein in two, but hemostasis was achieved laparoscopically in all cases. The procedure was converted to open surgery in three cases (5.0%). There was one Clavien-Dindo Grade 3 or worse postoperative complication (1 case of ileus, 1.7%). There were no surgery-related or in-hospital deaths.

Video 1 shows laparoscopic CME for cancer of the hepatic flexure of the transverse colon in a Type A/V patient, a comparatively easier procedure. Video 2 shows CVL during laparoscopic CME in a Type V/A patient with cancer of the hepatic flexure of the transverse colon. In this second patient, the first jejunal vein ran along the anterior surface of the SMA on its ventral side just caudal to the root of the middle colic artery (MCA), making the ligation of the MCA root extremely difficult. However, this procedure was made possible by passing some silicone tape around the SMV and pulling it ventrally and to the left.

## Discussion

This procedure can be performed comparatively safely. However, since it requires some skill, we consider that it should only be performed in suitable cases by teams with sufficient experience.

## Electronic supplementary material

Below is the link to the electronic supplementary material.

Supplementary video1 (AVI 1793267 kb)
